# Adrenal morphology and cortical function in patients with extrapulmonary tuberculosis: response to antituberculosis treatment

**DOI:** 10.20945/2359-4292-2021-0514

**Published:** 2024-02-29

**Authors:** Bashir Ahmad Laway, Bhanu Pratap Singh Blouria, Rafi Ahmad Jan, Moomin Hussain Bhat, Naseer Ahmad Choh

**Affiliations:** 1 Sher-I-Kashmir Institute of Medical Sciences Department of Endocrinology Srinagar Kashmir India Department of Endocrinology, Sher-I-Kashmir Institute of Medical Sciences, Srinagar, Kashmir, India; 2 Sher-I-Kashmir Institute of Medical Sciences General Medicine Srinagar Kashmir India General Medicine, Sher-I-Kashmir Institute of Medical Sciences, Srinagar, Kashmir, India; 3 Sher-I-Kashmir Institute of Medical Sciences Radio Diagnosis and Imaging Srinagar Kashmir India Radio Diagnosis and Imaging, Sher-I-Kashmir Institute of Medical Sciences, Srinagar, Kashmir, India

**Keywords:** Tuberculosis, extrapulmonary tuberculosis, endocrine, adrenal cortex, cortisol

## Abstract

**Objective:**

Enlargement of the adrenal glands and variable adrenocortical function have been reported in patients with pulmonary tuberculosis and, in a few studies, in patients with extrapulmonary tuberculosis (EPTB). However, none of the studies have evaluated the course of the adrenal morphology in these patients.

**Subjects and methods:**

Prospective study including 37 patients with EPTB and 37 healthy age- and sex-matched controls. The adrenal function was evaluated by measurement of cortisol levels at baseline and after stimulation with ACTH (Acton Prolongatum) before and 6 months after antituberculosis treatment. The size of both adrenal glands was evaluated using 64-slice computed tomography (CT) scanning before and 6 months after treatment. The findings were compared with those in a group of healthy matched controls.

**Results:**

Clinical and biochemical parameters were comparable between groups. The mean baseline serum cortisol level was significantly lower in the EPTB group (397.1 ± 184.9 nmol/L) compared with the control group (696.3 ± 101.8 nmol/L). Compared with controls, patients with EPTB had significantly lower mean cortisol levels at baseline and 1 hour after ACTH, both before (397 ± 184.9 nmol/L and 750.7 ± 176.8 nmol/L, respectively) and after (529.7 ± 100.4 nmol/L and 1017.2 ± 119.7 nmol/L, respectively) antituberculosis treatment. Both the length and thickness of the right and left adrenal glands were greater in patients with EPTB than in controls but became comparable to those in controls after treatment completion.

**Conclusions:**

Patients with EPTB have an enlarged adrenal size and low baseline and stimulated serum cortisol levels. After treatment completion, cortisol levels increased significantly, and the adrenal size normalized in these patients.

## INTRODUCTION

Tuberculosis has decreased in incidence in developed countries but remains a common cause of adrenal insufficiency in developing countries (1-3). Studies of adrenal reserve (using ACTH stimulation test) and adrenal size (using computed tomography [CT] scanning) in patients with tuberculosis have revealed conflicting results. While some have demonstrated a decreased adrenal reserve in 8%-58% of the patients with tuberculosis (4-6), some recent studies have shown a normal adrenal reserve in patients with active tuberculosis (7-9). Most of these studies have evaluated the adrenal size and/or functional reserve before antituberculosis treatment, but only a few have included a post-treatment evaluation (4,7). Additionally, data on the adrenal function and morphology in patients with extrapulmonary tuberculosis (EPTB) are limited. Some studies have included few patients with EPTB and analyzed their adrenal reserve (10,11), but none has evaluated both the adrenal size and its course after antituberculosis treatment.

Based on these considerations, the aim of this study was to evaluate the morphology and cortical function of the adrenal glands in patients with EPTB before and after antituberculosis treatment.

## SUBJECTS AND METHODS

### Study population

This prospective study was conducted from October 2018 to October 2020 and included 37 patients with EPTB aged 18-55 years. The patients were selected from the internal medicine outpatient services of a tertiary care hospital. A total of 37 age- and sex-matched healthy individuals were included as controls after undergoing thorough screening for adrenal insufficiency. The exclusion criteria were previous history of tuberculosis or antituberculosis treatment, clinical evidence of adrenal insufficiency, pregnancy, presence of autoimmune endocrine disease other than adrenal insufficiency, malignancy, and use of medications like steroids, oral contraceptives, antiepileptics, or ketoconazole.

The study was approved by the institutional ethics committee (IECSKIMS/2021-106. dated 30-7-2021), and all participants signed an informed consent form.

### Measurements

The participants underwent clinical examination, and fasting blood samples were collected at 8-9 a.m. for measurement of complete blood count, erythrocyte sedimentation rate (ESR), glucose, electrolytes (sodium and potassium), kidney and liver function tests, and serum cortisol. Depending on the clinical diagnosis of EPTB, other tests performed included the collection of morning urine or pleural, ascitic, pericardial, or cerebrospinal fluid samples for analysis of acid-fast bacilli (AFB), cartridge-based nucleic acid amplification test (CB-NAAT), and AFB culture. Fine-needle aspiration biopsy and cytology of lymph nodes, pleural or peritoneal tissue, or other abdominal enlargement were performed when necessary. Pulmonary tuberculosis was ruled out in all patients who had a normal chest X-ray and sputum testing negative for AFB after Ziehl-Neelsen staining.

### Diagnosis of extrapulmonary tuberculosis

The criteria used to diagnose EPTB included urine, pleural, pericardial, ascitic, or cerebrospinal fluid smear positive for AFB and/or CB-NAAT and/or culture positive for AFB or histopathological evidence of tuberculous granuloma. Since the yield of tubercle bacilli on smear, culture, or CB-NAAT is low in extrapulmonary sites, the response to antituberculosis treatment was considered the gold standard. As shown in Supplementary Table 1, all 37 patients with EPTB responded to antituberculosis treatment.

**Supplementary Table 1:** Diagnosis of tuberculosis in 37 patients of EPTB

S No	Diagnostic test	No of patients
1	Granulomatous inflammation pleural biopsy	9
2	Pleural fluid for CBNAAT	6
3	Pleural fluid positive for AFB	5
4	Pleural fluid with high ADA and response to ATT	4
5	Urine for AFB	4
6	CSF or AFB	3
7	Ascitic fluid for AB	3
8	Granulomatous inflammation lymph node	2
9	Aspirate positive for CBNAAT	1
	Total	37

### Assessment of adrenocortical function

A baseline venous sample was obtained from all patients and controls at 8-9 a.m. for measurement of cortisol level. All patients with EPTB underwent ACTH stimulation test with intramuscular injection of 25 U of ACTH (Acton Prolongatum, corticotrophin-carboxymethylcellulose BPC 73, Ferring Pharmaceuticals Pvt. Ltd., Thane, India) followed by venous blood draw at 60 minutes for measurement of cortisol level (12). The blood samples were allowed to settle for 15 minutes at room temperature before centrifugation. After centrifugation, the samples were stored at -80°C until assayed.

### Analysis

An automated hematology analyzer (Sysmex XT-2000i; Sysmex Corporation, Kobe, Japan) was used for complete blood count analysis. Biochemical measurements were performed using an automated analyzer (Beckman Coulter AU5800; Beckman Coulter Inc., Brea, CA, USA). Intra-assay and interassay variations were within the limits specified by the manufacturer. Serum cortisol levels were measured using chemiluminescent technology (UniCel DxI 800 Access Immunoassay Analyzer; Beckman Coulter) according to the manufacturer's recommendations.

### Adrenal imaging

The adrenal morphology was assessed using a 64-slice multidetector CT scanner (SOMATOM Sensation, Siemens Medical Solutions, Germany) with 3-mm slice thickness and reconstruction interval of 1.5-3 mm. The following adrenal dimensions were measured: maximum width (measured perpendicular to the long axis of the body of the gland at the junction of the gland's body and limbs), limb width (measured as the maximum thickness of the lateral and medial gland limbs perpendicular to the long axis of the limb), and length (measured as the cephalocaudal dimension of the gland). The length of the adrenal gland was calculated from the thickness of the number of sections in which the gland was visualized, *i.e.*, total craniocaudal length = number of sections multiplied by slice thickness (13,14). These measurements were taken at the time of diagnosis of EPTB (before treatment) and were repeated after completion of therapy. A single radiologist carried out all the measurements to prevent interobserver bias.

### Definition of adrenal insufficiency

Adrenal insufficiency was defined as the finding of a serum cortisol level below 500 nmol/L 1 hour after ACTH administration (12).

### Statistical analysis

The statistical analysis was performed using the software SPSS, version 20.0 (IBM SPSS Statistics for Windows, IBM Corp., Armonk, NY, USA). For normally distributed data, the results were expressed as mean ± standard deviation (SD), and for non-normally distributed data, the results were expressed as median and interquartile range. An independent samples t-test was used to compare mean (±SD) values between cases (EPTB group) and controls before antituberculosis treatment for normally distributed data; for non-normally distributed data, the Wilcoxon rank ligand test was used instead. Paired samples t-test was used to compare results in the patients’ group before and after treatment of tuberculosis. Spearman's correlation was used to analyze the relationship between levels of cortisol and blood glucose, sodium, and ESR before treatment. P values < 0.05 were considered significant.

## RESULTS

The study included 37 patients with EPTB and 37 age- and sex-matched healthy controls. The EPTB group consisted of patients with tuberculous pleural effusion (n = 19), urogenital tuberculosis (n = 4), tuberculous meningitis (n = 3), tuberculous ascites (n = 3), disseminated tuberculosis (n = 3), tuberculous pleural effusion (n = 2), tuberculous lymphadenitis (n = 2), and tuberculous spinal abscess (n = 1).

### Comparison of serum cortisol and adrenal size between groups

Both groups were comparable in age, body mass index, and levels of hemoglobin and potassium. In contrast, the EPTB group had significantly higher ESR and significantly lower sodium and glucose levels than the control group. Mean baseline cortisol levels were within the normal range in both groups but were significantly lower in the EPTB group (397 ± 184.9 nmol/L, range 90.2-730.3 nmol/L) compared with the control group (696.1 ± 101.8 nmol/L, range 571.6-833.2 nmol/L; p = 0.001) (Table 1). Additionally, the adrenal size was significantly larger in the EPTB compared with the control group.

**Table 1:** Clinical and biochemical parameters in patients with extrapulmonary tuberculosis compared with controls

Parameter	EPTB group	Control group	P value
Age (years)*	35 (19.5-65.0)	40 (22.0-58.0)	0.615
BMI (kg/m^2^)	20.4 ± 3.3	21.2 ± 0.9	0.124
SBP (mmHg)	118.4 ± 11.3	119.5 ± 4.2	0.540
DBP (mmHg)	76 ± 7.7	74.7 ± 7	0.431
Hb (g/dL)	11.1 ± 2.2	10.5 ± 1.1	0.118
ESR (mm/hour)	36.5 ± 7.9	5.9 ± 2	0.000
Plasma glucose (mmol/L)	4.7 ± 0.3	7.2 ± 1.1	0.000
Serum sodium (mmol/L)	134.4 ± 3.4	137 ± 3.5	0.001
Serum potassium (mmol/L)	3.6 ± 0.4	3.6 ± 0.2	0.594
Baseline cortisol (nmol/L)	397.1 ± 184.9	696.3 ± 101.8	0.001
Baseline cortisol (nmol/L)**	90.2-730.3	571.6-833.2	0.001

Abbreviations: BMI, body mass index; DBP, diastolic blood pressure; EPTB, extrapulmonary tuberculosis; ESR, erythrocyte sedimentation rate; Hb, hemoglobin; SBP, systolic blood pressure. The values are shown as means ± standard deviations

* medians and interquartile ranges

** range.

### Comparison of serum cortisol and adrenal size before and after treatment

In patients with EPTB, the mean baseline and stimulated serum cortisol increased significantly after treatment completion (p = 0.002 for both). None of the patients had adrenal insufficiency (*i.e.*, cortisol level < 500 nmol/L after ACTH stimulation) before or after EPTB treatment (Table 2). Compared with healthy controls, patients with EPTB had significantly longer adrenal length and increased thickness of the adrenal limbs on both the right and left glands before treatment (Table 3). A repeat CT after the completion of antituberculosis treatment revealed a significant decrease in adrenal dimensions (Table 4). There was no significant correlation between serum levels of baseline cortisol and glucose (r = 0.06, p = 0.694), sodium (r = 0.120, p = 0.479), or ESR (r = 0.26, p = 0.119) before EPTB treatment.

**Table 2:** Comparison of baseline and stimulated cortisol in patients with extrapulmonary tuberculosis before and after treatment

Parameter	Before treatment	After treatment	P value
Baseline cortisol (nmol/L)	397 ± 184.9	529.7 ± 100.4	0.002
Stimulated cortisol (nmol/L)	750.7 ± 176.8	1017.2 ± 119.7	0.002
Adrenal insufficiency	Nil	Nil	–

The values are shown as means ± standard deviations.

**Table 3:** Adrenal dimensions (mm) in patients with extrapulmonary tuberculosis before treatment compared with controls

Dimension (mm)	EPTB group		Control group		P value
	Right	Left	Right	Left	
Width	5.8 (5.2-6.0)	5.6 (5.3-6.4)	5.9 (5.8-6)	6.8 (6.7-6.9)	<0.01
Length	13 (12-14.9)	13.4 (12.1-14.5)	28.7 (28.5-28.8)	28.4 (27.8-28.6)	<0.01
Lateral limb	4.2 (3.1-4.8)	4.4 (3.5-4.8)	2.8 (2.7-2.8)	2.9 (2.8-3.0)	<0.01
Medial limb	4.1 (3.5-4.6)	4.2 (3.6-4.3)	2.6 (2.5-2.8)	3.4 (3.2-3.4)	<0.01

The values are shown as medians and interquartile ranges.

**Table 4:** Adrenal gland dimensions (mm) before and after treatment of extrapulmonary tuberculosis

Dimension (mm)	Before treatment		After treatmen		P value
	Right	Left	Right	Left	
	5.8 (5.2-6.0)	5.6 (5.3-6.4)	5.2 (4.8-5.6)	5.1 (4.6-5.6)	0.001
Length	13 (12-14.9)	13.4 (12.1-14.5)	12.2 (11.2-13.1)	12.6 (11.8-13.5)	0.001
Lateral limb	4.2 (3.1-4.8)	4.4 (3.5-4.8)	3.9 (2.6-4.4)	3.9 (2.8-4.3)	0.002
Medial limb	4.1 (3.5-4.6)	4.2 (3.6-4.3)	3.8 (2.8-4)	3.9 (3.3-4.1)	0.001

The values are shown as medians and interquartile ranges.

## DISCUSSION

### Adrenocortical function before and after antituberculosis treatment

We found a lower baseline serum cortisol in patients with EPTB compared with controls, although none had adrenal insufficiency. We also found a significant increase in baseline and stimulated cortisol after antituberculosis treatment completion. None of the women in the control group was pregnant or taking estrogen during the study.

Many studies have demonstrated overt or so-called "subclinical" adrenal insufficiency in patients with pulmonary tuberculosis (6,10,15,16). These studies have mainly used the yardstick of the cortisol increase over baseline after the short Synacthen test (SST), a criterion that has not been widely accepted (17,18). In contrast, some recent studies in patients with pulmonary tuberculosis found no evidence of adrenal insufficiency based on an absolute cortisol value > 500 nmol/L after the SST (7-9).

A few well-designed studies (some including a control group) have found normal baseline and stimulated cortisol levels in patients with chronic pulmonary tuberculosis or EPTB (4,7,8). Barnes and cols. studied patients with pulmonary tuberculosis (n = 30), EPTB (n = 30), and miliary tuberculosis (n = 30) and found a normal baseline cortisol in 70% of them and a normal cortisol increase of > 198.65 nmol/L in 30% of the patients with EPTB. Of note, the study did not include a control group for comparison (4). Kelestimur and cols., in a study of patients with acute pulmonary tuberculosis (n = 20), chronic pulmonary tuberculosis (n = 41), and healthy controls (n = 20), found that cortisol levels at baseline and 30 and 60 minutes after SST were higher in patients with acute pulmonary tuberculosis than in those with chronic pulmonary tuberculosis, but observed no difference between patients with chronic pulmonary tuberculosis and healthy controls (8). No patients with EPTB were included in the study.

After completion of antituberculosis treatment in the present study, there was a significant increase in cortisol levels both at baseline and 60 minutes after ACTH compared with pretreatment values. Studies of cortisol dynamics in patients with EPTB are limited (4,10,11), and only two have included patients with EPTB after antituberculosis treatment. In the study by Barnes and cols., baseline cortisol was normal in 70% of the patients with EPTB (n = 30), and this percentage increased to 96% at 3-4 weeks after the start of antituberculosis treatment (4). In an Indian study, Sharma and cols. evaluated the adrenal reserve of 105 patients with tuberculosis (including 33 patients with EPTB) using a standard ACTH stimulation test at baseline and followed the patients every 6 months for 30 months. At baseline, almost half of the patients had impaired adrenal response. The percentage of responders increased to 71% at 6 months and to 97% at 24 months (16). Our results are aligned with those from the Sharma and cols. study in the sense that serum cortisol concentration was initially low (albeit within the normal range) and increased with EPTB treatment.

Tuberculosis affects the adrenal cortex in two ways. One is by increasing cortisol production due to heightened activity of the hypothalamic-pituitary-adrenal (HPA) axis (in active tuberculosis), and the other is by reducing cortisol production due to a direct involvement of the adrenal cortex (19). Whether the hypercortisolemia due to activation of HPA axis normalizes after tuberculosis treatment remains unknown. There is sufficient evidence indicating that relatively low cortisol production in patients with pulmonary tuberculosis or EPTB improves significantly and normalizes after treatment of tuberculosis (4,16). We believe that relatively low initial cortisol production in patients with EPTB is due to mild adrenalitis (which results in some impairment of the adrenocortical function that is not severe enough to cause adrenal insufficiency) and that this phenomenon reverses after EPTB treatment (7).

### Adrenal size before and after tuberculosis treatment

The present study showed that the size of the adrenal glands was increased in patients with EPTB compared with healthy controls and that the size normalized after 6 months of antituberculosis treatment. Only a few authors have examined the adrenal morphology by measuring the size of the adrenal gland using noncontrast CT. All these studies reported an increased adrenal size at the time of the active infection (7,8,17,20). There are two reasons for adrenal enlargement in patients with tuberculosis, one is due to adrenal hyperplasia associated with increased vascularity in acute tuberculosis (21), and the other is due to adrenalitis associated with increased gland size and decreased gland function (7). We found normalization of the size of enlarged adrenals in patients with EPTB after treatment of tuberculosis. Two previous smaller studies have also reported normalization of the adrenal size after tuberculosis treatment (17,20). We have previously reported that adrenal enlargement in patients with pulmonary tuberculosis (n = 45) reversed after tuberculosis treatment (7). The adrenal size and its course after EPTB treatment had not been previously studied. In the present study, patients with EPTB showed adrenal enlargement that reversed after antituberculosis treatment. This adrenal response appears similar to that observed in patients with pulmonary tuberculosis.

### Limitations

Since Synacthen is not available in India, Acton Prolongatum was used instead. However, this test has been sufficiently validated in India (12). Also, ACTH-stimulated cortisol levels were not available in the control group for comparison with the EPTB group. Because the study included consecutive patients with EPTB and the sample size was not calculated, the possibility of a beta error cannot be excluded.

In conclusion, we conclude that, compared with controls, patients with EPTB have low baseline and stimulated serum cortisol levels and adrenal enlargement, both of which reverse after antituberculosis treatment.
